# Evaluation of non-exudative microcystoid macular abnormalities secondary to retinal vein occlusion

**DOI:** 10.1007/s00417-021-05250-9

**Published:** 2021-06-22

**Authors:** Anibal Francone, Andrea Govetto, Lisa Yun, Juliet Essilfie, Kouros Nouri-Mahdavi, David Sarraf, Jean-Pierre Hubschman

**Affiliations:** 1 Retina Division, Stein Eye Institute, University of California Los Angeles, Los Angeles, CA USA; 2 Ophthalmology Department, Fatebenefratelli-Oftalmico Hospital, Milan, Italy; 3 Glaucoma Division, Stein Eye Institute, University of California Los Angeles, Los Angeles, CA USA; 4 David Geffen School of Medicine At UCLA, Retinal Disorders and Ophthalmic Genetics Division, Stein Eye Institute, Los Angeles, CA USA; 5 Greater Los Angeles VA Healthcare Center, Los Angeles, CA USA

**Keywords:** Microcystoid macular abnormalities, Retinal vein occlusion, Central retinal vein occlusion, Branch retinal vein occlusion, Glaucoma, Optic neuropathy, Microcystoid macular changes, Microcystoid degeneration, Microcysts, Inner nuclear layer, Optical coherence tomography

## Abstract

**Purpose:**

We aimed to investigate non-exudative microcystoid macular abnormalities for visual and anatomical outcome in patients with retinal vein occlusion (RVO) with and without glaucomatous optic neuropathy (GON).

**Methods:**

Medical records of 124 eyes (105 patients) with RVO were reviewed and analyzed. Eyes demonstrating microcystoid macular abnormalities were divided into 2 groups, those with evidence of glaucoma (group A) and those without glaucoma (group B). Best-corrected visual acuity (BCVA), the prevalence and number of microcystoid macular abnormalities, and number of intravitreal anti-vascular endothelial growth factor (anti-VEGF) injections were compared at baseline and follow-up.

**Results:**

Seventy-one out of 105 eyes (67.6%) with RVO displayed microcystoid macular abnormalities. Thirty-eight out of 71 eyes (53.5%) presented with concomitant glaucoma (group A), while the remaining 33 eyes (42.6%) had no history of glaucoma (group B). At the end of the follow-up period, mean BCVA was worse in group A versus group B (20/80 versus 20/40, respectively; p = .003). The mean number of anti-VEGF injections was 10.1 ± 9.2 in group A versus 5.9 ± 6.9 in group B (p = .03).

**Conclusion:**

Eyes with RVO and concomitant glaucoma exhibited a significantly higher number of microcystoid macular abnormalities and worse BCVA versus eyes with RVO without glaucoma.

## Introduction


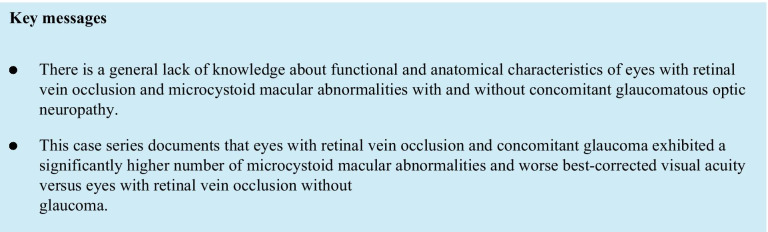


The most common cause of vision loss in eyes with retinal vein occlusion (RVO) is cystoid macular edema (CME) [1, 2]. Recent pivotal clinical trials have demonstrated the efficacy of intravitreal anti-VEGF therapy which is associated with significant improvement of visual acuity and remarkable reduction or resolution of CME in eyes with RVO [3–7].

Traditionally, the gold standard diagnosis of exudative CME has required the detection of petaloid leakage with fluorescein angiography (FA) [8]. Spectral-domain optical coherence tomography (SD-OCT) is a more advanced imaging modality that can readily display cysts within the inner nuclear (INL) and outer plexiform layers (OPL) of the retina. CME morphology may differ according to its location: in the inner nuclear layer (INL) CME manifests as small, regular, and roundish cystoid cavities, while in the outer nuclear layer (ONL), it usually presents with larger and more irregular cystoid cavities [9].

More recently, non-exudative microcystoid degeneration of the INL has been described using SD-OCT imaging and may be confused with exudative CME [10]. This abnormality has been associated with various forms of optic neuropathy [11, 12] including 3 to 6% of eyes with glaucomatous optic neuropathy (GON) [13, 14] and 55% of eyes with combined epiretinal membrane and GON [15]. These microcysts, also referred to as retrograde maculopathy [16] or pseudocysts [17], may have a slit like or elliptical morphology in the INL with SD-OCT, fail to display leakage with FA, and may represent a form of Muller cells degeneration, the cell bodies of which reside in the INL. [18].

Systemic vascular disease and ocular conditions such as glaucoma are well-established risk factor for RVO, [19, 20] as the association between RVO and glaucoma has been described in previous studies [21, 22]. Since glaucoma and RVO often coexist, the appearance of microcystoid macular abnormalities may be misinterpreted and complicate the decision-making of the disease management and the prediction of treatment response.

The objectives of this study were to compare functional and anatomical characteristics of eyes with retinal vein occlusion and microcystoid macular abnormalities with and without preexisting glaucomatous optic neuropathy.

## Methods

### Study participants

Consecutive patients diagnosed with RVO between January 1, 2008, and August, 31 2017, in the Retina Division at the Stein Eye Institute, University of California Los Angeles were identified and included in this retrospective chart review. All data for this study were collected and analyzed in accordance with the policies and procedures of the Institutional Review Board of the University of California Los Angeles and adhered to the tenets of the Declaration of Helsinki and the Health Insurance Portability and Accountability Act (HIPAA).

### Data collection

Cases were identified by a medical billing record search, using the International Classification of Diseases Ninth Revision (ICD-9) diagnosis code 362.36 for branch retinal vein occlusion (BRVO) and 362.35 for central retinal vein occlusion (CRVO). Patients were included if they met criteria for unilateral or bilateral BRVO or CRVO with cystoid macular edema — mean central subfield thickness ≥ 250 µm — and a minimum follow-up of 12 months. Exclusion criteria included retinal detachment, severe ocular trauma, uveitis, epiretinal membrane, macular diseases, diabetic retinopathy, ocular surgery — including uneventful cataract extraction — in the last 12 months, previous pars plana vitrectomy, endophthalmitis, and neurological disorders. In patients in whom both eyes were eligible, one eye was included at random. Paper and electronic records of the enrolled patients were reviewed to identify patients also diagnosed with preexisting primary open-angle glaucoma (POAG) (code 365.11). The diagnosis of glaucoma was confirmed by a glaucoma specialist based on glaucomatous damage to the optic disc (retinal nerve fiber layer thickness) and abnormal visual field. The severity of the GON was graded based on visual fields as mild, moderate, or severe according to the Hodapp-Parrish-Anderson scale [23].

Demographic and clinical data were extracted from records. Best-corrected visual acuity (BCVA) at presentation and over the follow-up period was recorded in Snellen units and was converted to logarithm of the minimal angle of resolution (logMAR) for statistical analysis.

### Optical coherence tomography analysis

**Fig. 1:**
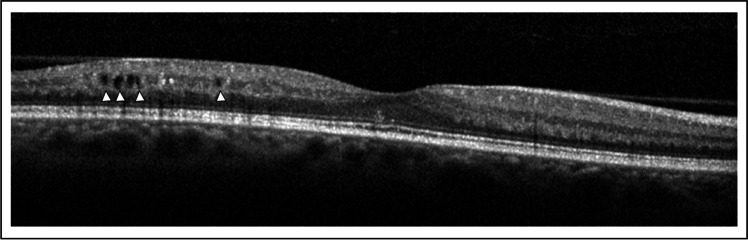
Morphology of non-exudative microcystoid macular abnormalities in retinal vein occlusion. Non-exudative microcystoid macular abnormalities were defined with optical coherence tomography as multiple, small, slit like, ovoidal, or ellipsoid hyporeflective spaces located in the inner nuclear layer at the parafoveal area (white arrowheads)

**Fig. 2:**
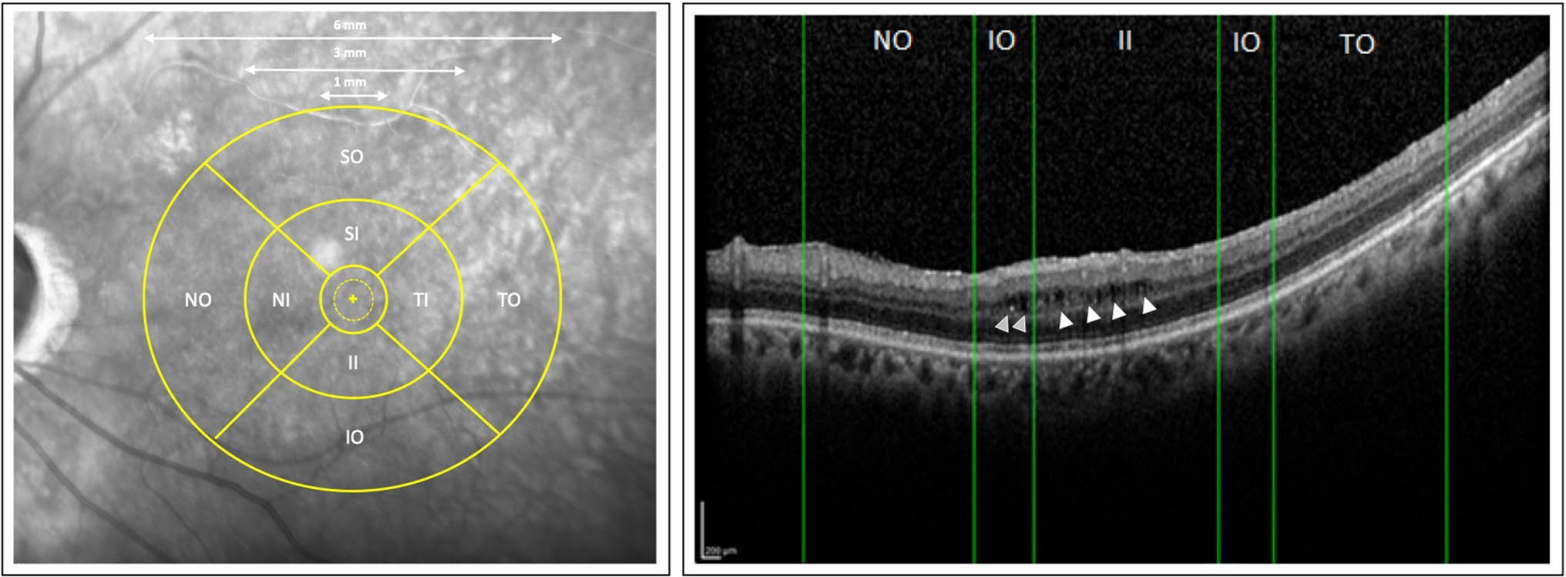
Heidelberg Eye Explorer: The Early Treatment Diabetic Retinopathy Study (ETDRS) circle grid overlay and spectral-domain optical coherence tomography. (Left) The Early Treatment Diabetic Retinopathy Study grid consist of 3 concentric circles centered on the fovea that divide the macula into 3 zones; the fovea (less than 1 mm diameter), the inner macula (1 to 3 mm), and the outer macula (3 to 6 mm). These 3 concentric circles delimited 9 macular regions: superior outer (SO), temporal outer (TO), inferior outer (IO), nasal outer (NO), superior inner (SI), temporal inner (TI), inferior inner (II), and nasal inner (NI) regions. (Right) Green vertical lines show the boundaries of the Early Treatment Diabetic Retinopathy Study regions on the spectral-domain optical coherence tomography B-scan. Scrolling through the entire scan and guided by the overlay, we counted the number of microcystoid macular abnormalities in each region. Microcystoid macular abnormalities are shown in the inferior outer (grey arrowheads) and in the inferior inner (white arrowheads) regions

Spectralis OCT (Heidelberg Engineering GmbH, Heidelberg, Germany) testing was performed using the 20 × 15-degree macula raster with 19 horizontal B-scans spaced 242 mm. SD-OCT images were reviewed with the Heidelberg Eye Explorer (version 1.8.6.0) using the HRA/Spectralis Viewing Module (version 5.8.3.0). As illustrated in Fig. 1, non-exudative microcystoid macular abnormalities were defined as multiple, small hyporeflective roundish-elliptical cystoid cavities, without walls, located in the INL with SD-OCT and not confluent with cystoid cavities in other retinal layers including the OPL [10]. Two independent, trained and masked observers (L. Y. and A. F.) evaluated the presence of microcystoid macular abnormalities in all SD-OCT scans. The OCT circle grid overlay from the Early Treatment Diabetic Retinopathy Study (EDTRS) was placed over infrared images (IR) centered at the fovea. The EDTRS grid comprised 3 concentric circles that divided the macula into 3 zones; the fovea (less than 1 mm diameter), the inner macula ring (1 to 3 mm), and the outer macula ring (3 to 6 mm). As shown in Fig. 2, these 3 concentric circles delimited 9 macular regions that were labeled as fovea (F), superior outer (SO), temporal outer (TO), inferior outer (IO), nasal outer (NO), superior inner (SI), temporal inner (TI), inferior inner (II), and nasal inner (NI) regions [24]. At each visit, two independent masked observers (J.E. and A.F.) manually counted the total number of microcystoid macular abnormalities in each horizontal B-scan within the boundaries of each area. The arithmetic mean of the values from the main reader (A.F.) was used for the analysis, whereas those from the second reader (J. E.) were only used to calculate the intergrader agreement. In addition, mean central foveal thickness (CFT) recorded from the automatic ‘‘thickness map’’ calculation of the Heidelberg Eye Explorer.

### Statistical analysis

All data analyses were performed using SPSS software version 25 (SPSS, Inc., Chicago, Illinois, USA). The primary outcome was the number of microcystoid macular abnormalities in the inner nuclear layer in eyes with RVO with or without preexisting glaucomatous optic neuropathy. The secondary outcome were the change in best corrected visual acuity (BCVA) from baseline to last follow-up and the number of intravitreal injections received. Snellen visual acuity was converted to the logarithm of the minimal angle of resolution (logMAR) units. Data were entered into a Microsoft Excel 2010 (Microsoft Corporation, Redmond, WA, USA) datasheet for tabulation and descriptive statistics. The procedure for determining optimal sample in this retrospective chart review was the level of precision in the estimation of the primary outcome and for intragroup comparisons to achieve a level of statistical significance (5%) with a power of 80%. Shapiro–Wilk test was used for testing normality. Parametric and nonparametric tests (Mann–Whitney U, Wilcoxon signed rank test) were used to compare quantitative variables. Categorical variables were analyzed using chi-square or Fisher’s exact tests. Intraclass correlation coefficients with 95% confidence intervals were calculated to evaluate intergrader agreement.

## Results

### Baseline characteristics of the study population

A total of 124 eyes from 105 patients with RVO and macular edema, of which 41 (39%) were males and 64 (61%) were females, were identified with a mean age of 75.9 years ± 14.8 (range 36–97 years). Nineteen out of 105 patients (18.1%) presented bilateral RVO, one eye per individual was chosen; therefore 105 eyes of 105 subjects were included for analysis.

Seventy-one out of 105 eyes (67.6%) showed non-exudative microcystoid macular abnormalities with a mean number of 44.1 ± 48 per eye. Of these 71 eyes, 38 (53.5%) were diagnosed with concomitant glaucoma (group A), while 33 eyes (46.5%) did not have glaucoma (group B). In group A, 20 out of 38 eyes (52.6%) had CRVO, while the remaining 18 eyes (47.4%) had BRVO. In group B, CRVO and BRVO were evenly distributed among the study population (16 out of 33 eyes (48.5%) and 17 out of 33 eyes (51.5%) for both conditions, respectively). Previous therapies for CME, glaucoma staging, and treatment are presented in Table 1.

**Table 1:** Baseline characteristics of the study population

Group (Number of eyes with microcystoid macular abnormalities)	RVO (clasiffication)	History of RVO treatment	Glaucoma (Stage)	Topical antiglaucoma drops	Antiglaucoma surgery
Glaucoma (n = 38)	CRVO: 20/38 (52.6%) BRVO: 18/38 (47.4%)	Anti-VEGF: 9/38 (23.7%) FML: 3/38 (7.9%) No treatment: 26/38 (68.4%)	Mild: 16/38 (42.1%) Moderate: 11/38 (28.95%) Severe: 11/38 (28.95%)	PGA: 11/38 (28.9%) T + D: 22/38 (57.9%) T + B: 12/38 (31.6%)	Trab: 6/38 (15.8%) Tube-Shunt: 5/38 (13.2%) PI: 3/38 (7.9%) SLT: 4/38 (10.5%) Micro-Stent: 1/38 (2.6%)
Non-glaucoma (n = 33)	CRVO: 16/33 (48.5%) BRVO: 17/33 (51.5%)	Anti-VEGF: 5/33 (15.1%) DEX implant: 2/33 (6.1%) No treatment: 26/33 (78.8%)	N/A	N/A	N/A
*P* value	0.8^a^	0.6^a^			

*RVO* retinal vein occlusion; *CRVO* central retina vein occlusion; *BRVO* branch retinal vein occlusion; *anti-VEGF* anti-vascular endothelial growth factor; *DEX* dexamethasone; *FML* focal macular laser; *PGA* prostaglandin analogue; *T* + *D* timolol + dorzolamide; *T* + *B* timolol + brimonidine; *Trab* trabeculectomy; *PI* peripheral iridotomy; *SLT* selective laser trabeculoplasty ^a^Fisher’s exact test

### Baseline associations of microcystoid macular abnormalities

The mean BCVA was significantly lower in group A compared to group B (20/125 vs 20/80, respectively; *P* = 0.003). The mean number of non-exudative microcystoid macular abnormalities was significantly different among the two subgroups, being higher in group A compared to group B (27.4 ± 31.5 and 16.1 ± 14.3, respectively; *P* = 0.03). In addition, the mean CFT measurement was significantly thicker in the glaucomatous group and was compared with the non-glaucomatous group (431.6 ± 53 µm and 391.5 ± 51 µm, respectively; *P* = 0.04).

### Follow-up and treatment

The mean follow-up was similar in both groups (4.2 ± 2.8 and 4.6 ± 2.2 years, respectively (*P* = 0.83). At the end of the follow-up period, the mean BCVA was worse in group A than group B (20/80 vs. 20/40, respectively; *P* = 0.003).

**Fig. 3:**
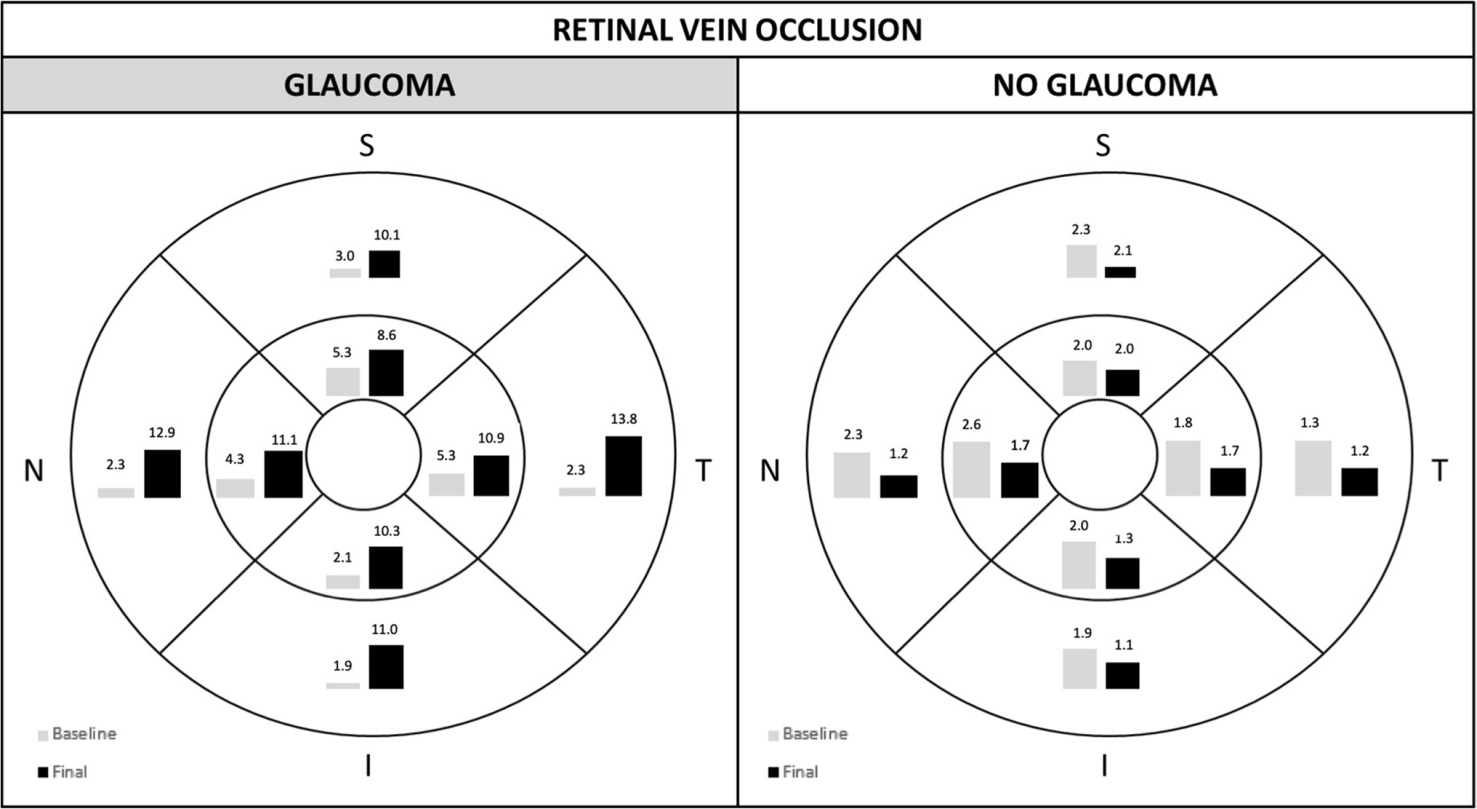
Mean number of microcystoid macular abnormalities by Early Treatment Diabetic Retinopathy Study (EDTRS) region at baseline and at the end of the follow-up period. (Left) At the end of the follow up period, eyes from the glaucoma group presented a significantly increased in the mean number of microcystoid macular abnormalities in the 8 EDTRS areas. The increase was proportionally greater in the outer ring compared to the inner ring, showing a centrifugal dispersion. (Right) Eyes from the non-glaucoma group demonstrated a lower mean number of microcystoid macular abnormalities at presentation compared to the glaucoma group and a generalized reduction at the end of the follow-up compared to baseline

Non-exudative microcystoid macular abnormalities were found in 17 eyes (17/38, 44.7%) in the glaucoma group and in 3 eyes (5/33, 15.2%) in the non-glaucoma group at the final visit. As illustrated in Fig. 3, the mean number of microcystoid macular abnormalities increased in each of the 8 EDTRS areas evaluated in the glaucoma group (total mean number of microcystoid macular abnormalities: 88.7 ± 15.1 *P* = 0.025), whereas it decreased in each of the 8 EDTRS areas evaluated in group B (total mean number of microcystoid macular abnormalities: 11.2 ± 10.4; *P* = 0.02) compared to baseline. In the group A, the increase was proportionally greater in the outer ring in comparison with the inner ring (4.9 versus 2.5 times, respectively). The outcome from the logistic regression model demonstrated that eyes with moderate glaucoma presented an significantly increased likelihood to develop non-exudative microcystoid macular abnormalities (*P* = 0.02). There was an excellent agreement in measurements between the two readers with a correlation coefficient of 0.985 (95% confidence interval, 0.984–0.986).

Mean CFT decreased significantly in both glaucomatous and non-glaucomatous groups (351.3 ± 93 µm and 273.7 ± 45.2 µm, respectively; *P* = 0.002). However, mean average reduction in CFT was greater in the non-glaucoma group than in the glaucoma group (97.8 ± 111.2 μm and 57.1 ± 185.2 μm, respectively; *P* = 0.048).

The total mean number of anti-VEGF injections was significantly greater in group A than in group B (10.1 ± 9.2 versus 5.9 ± 6.9; *P* = 0.03). In eyes with CRVO, the mean total number of injections was higher in group A compared to group B (11.2 ± 10.7 and 5.4 ± 3.9, respectively; *P* = 0.035). Similarly, in eyes with BRVO, the number of injections was also higher in group A versus group B (9.1 ± 7.3 versus 3.8 ± 3.5; *P* = 0.04).

In group A, the mean number of injections was 9.9 ± 7.3 in eyes with mild glaucoma, 12.2 ± 9.3 in eyes with moderate glaucoma, and 7.1 ± 6.9 in eyes with the severe stage of glaucoma; the difference among these subgroups was not statistically significant (*P* = 0.3). No severe complications from intravitreal injections were observed. Functional and anatomical results are summarized in Table 2.

**Table 2:** Functional and Anatomic Results in RVO Eye

Group (Number of Eyes)	Mean follow-up (years)	BCVA (logMar)			Number of anti-VEGF injections	Microcystoid macular abnormalities (mean ± SD)		Mean CFT reduction: baseline-final (μm)
		Baseline	Final			Baseline	Final	
Glaucoma (n = 38)	4.2 ± 2.8	0.8 ± 0.4	0.6 ± 0.1	10.1 ± 9.2		27.4 ± 31.5	88.7 ± 15.1	57.1 ± 185.2
No glaucoma (n = 33)	4.6 ± 2.2	0.6 ± 0.2	0.3 ± 0.3	5.9 ± 6.9		16.1 ± 14.3	11.2 ± 10.4	97.8 ± 111.2
*P* value	0.83	0.003	0.003	0.03		0.03	0.02	0.048

*BCVA* best-corrected visual acuity; *CFT* central foveal thickness; *logMAR* logarithm of minimal angle of resolution

## Discussion

This study reports the presence and course of non-exudative microcystoid macular abnormalities in eyes with RVO as a function of presence of glaucoma. After a detailed analysis of baseline OCT scans, microcystoid macular abnormalities were identified in 71 eyes (67.6%) with underlying CME related to RVO, a prevalence that is significantly higher compared to previous work. Published studies have reported that microcystoid degeneration occurs in 6% of eyes with GON, 9% to 20% of eyes with non-GON, and up to 55% in eyes with ERM and GON [13, 15, 16, 25]. However, identification of non-exudative microcystoid macular abnormalities may be very challenging as these lesions often comprise the spectrum of exudative or inflammatory CME. The lack of petaloid leakage with fluorescein angiography can be an important clue or biomarker of non-exudative microcystoid degeneration [18]. However, FA is an invasive technique that requires the injection of dye and is prohibitive to use on a repeated basis. Moreover, leakage from other sources such as an impaired retinal pigment epithelium may also confound diagnosis of CME [26]. SD-OCT identification of slit like or elliptical hyporeflective lesions in the INL may provide an alternate and precise means of diagnosis of non-exudative microcystoid degeneration.

In the present study, SD-OCT images illustrated that the architecture of microcystoid macular abnormalities were comparable in eyes with and without glaucoma. Although the pathophysiology of microcystoid macular abnormalities remains unclear, there is a general agreement that Muller cell dysfunction may play a role [18, 27, 28]. As described by Reichenbach, [29] Muller cells may display osmotic swelling of their cell bodies in conditions clinically associated with macular edema including ischemia and ocular inflammation [30, 31]. Vascular leakage may further promote the development of microcystoid macular abnormalities [32].

**Fig. 4:**
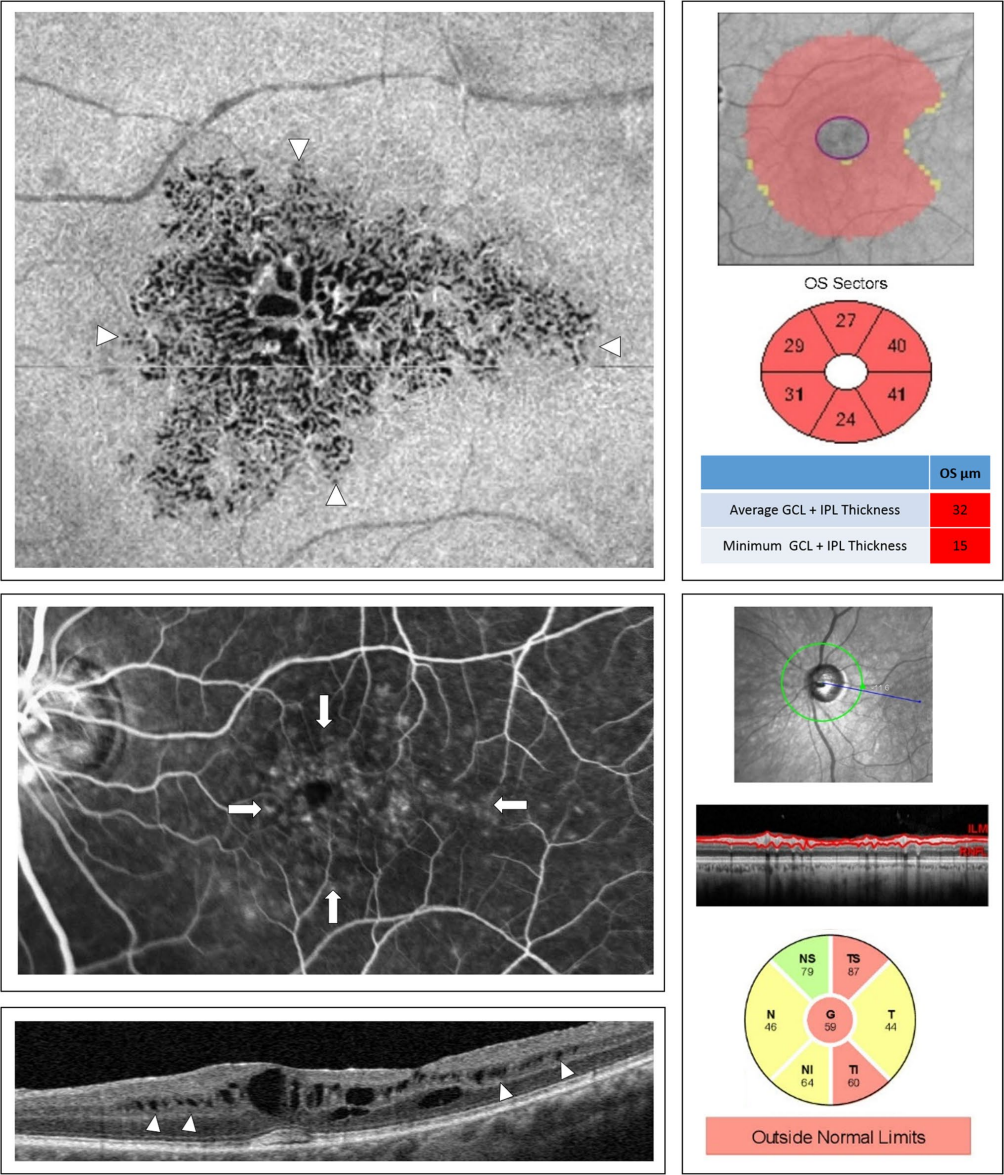
Multimodal imaging of non-exudative microcystoid macular abnormalities. This eye with branch retinal vein occlusion was diagnosed with moderate glaucoma. (Top left) The en-face segmentation of the inner nuclear layer (INL) reveals multiple hyporeflective cystoid spaces surrounding the foveal area (white arrowheads).(Middle left) Leakage on fluorescein angiography (42 s) does not seem to correlate with the extension of the microcystoid macular abnormalities (white arrows). (Bottom left) Microcystoid macular abnormalities (white arrowhead) on spectral-domain optical coherence tomography B-scan. (Top right) Macular ganglion cell analysis (GCA) revealed extensive ganglion cell layer thinning. (Bottom right) Measuring peripapillary retinal nerve fiber layer thickness (RNFLT) with spectral-domain optical coherence tomography demonstrated evidence of significant glaucomatous optic nerve damage

Abegg [16] speculated that optic neuropathies, like glaucoma, may trigger a retrograde transcellular Muller cell degeneration that impairs fluid absorption from the retina and may, therefore, cause microcystoid macular abnormalities [16]. Hence, retinal vein occlusion may cause intraretinal fluid accumulation as a result of the breakdown of the inner blood-retinal barrier and impairment of Muller cell function which may be further exacerbated by GON [27, 33]. Inner blood-retinal barrier breakdown and macular ischemia in conjunction with retrograde axonal injury may result in augmented insult to Muller cell function. This may explain the higher prevalence of microcystoid macular abnormalities at the end of the follow-up in the glaucoma group versus the non-glaucoma group (44.7% versus 15.2%, respectively). Furthermore, in group A the number of microcystoid macular abnormalities was considerably higher in the inner circle at baseline, whereas at the end of the follow-up, it increased in each of the 8 regions of the ETDRS grid, with a proportionally greater increased in the outer ring versus the inner ring. As demonstrated by multimodal imaging in Fig. 4, this centrifugal migration may be facilitated by permanent damage of the inner retinal layers present in the retina of patients with glaucoma that would make the cellular structure more vulnerable [34].

Sebag [35] has suggested that in retinal vascular diseases, the prevalence of vitreomacular adhesion may be more common versus in age-matched controls and the incidence of macular edema may be significantly higher in eyes with vitreomacular attachment compared to eyes with vitreomacular separation. Anterior vitreomacular traction (VMT) may result in microcystoid foveal degeneration located in the INL in the absence of capillary leakage [32]. This mechanical stress should also be considered part of the mechanism by which microcystoid macular abnormalities develop. The OCT images in this study did not provide adequate information to examine and determine the potential role of vitreous traction in the pathophysiology of microcystoid macular abnormalities. Future studies that include the use of enhanced vitreous imaging OCT may provide critical insight regarding the vitreoretinal interface that may or may not contribute to the development of microcystoid degeneration.

**Fig. 5:**
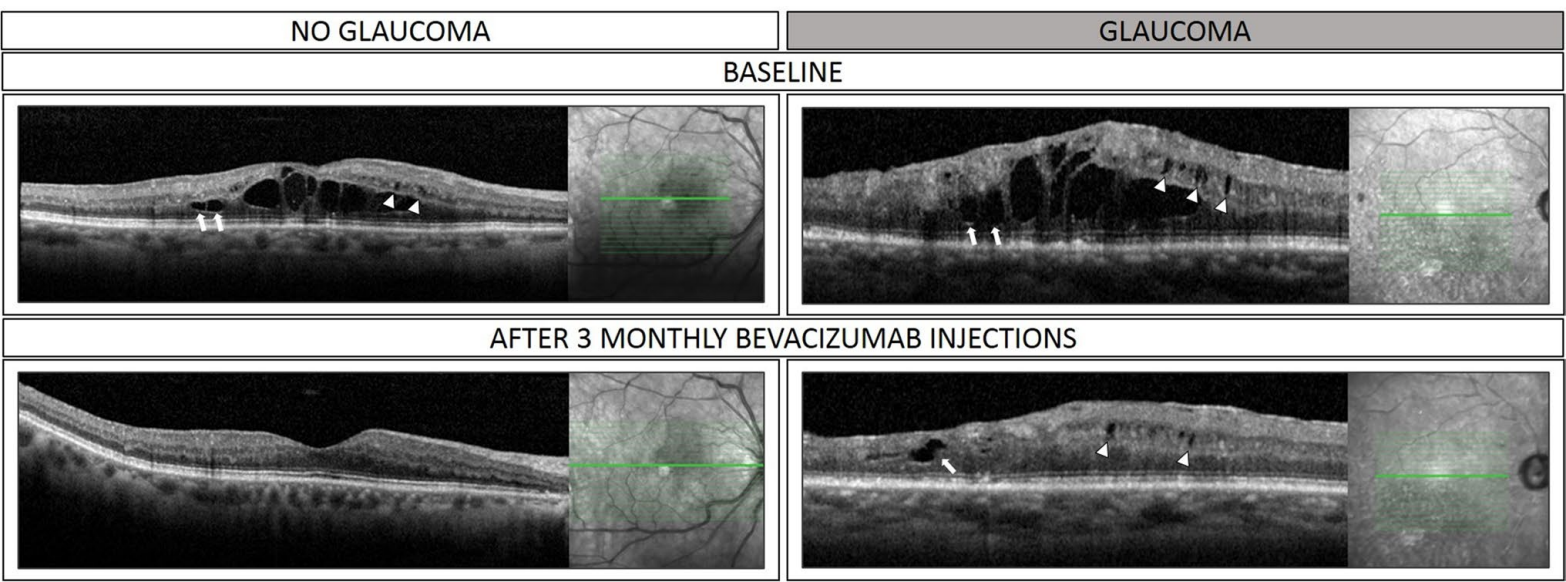
Morphologic changes over the follow-up period in eyes with and without glaucoma. (Top left) Superior branch retinal vein occlusion without glaucoma. At baseline, optical coherence tomography scan shows microcystoid macular abnormalities (white arrowheads) and pseudocysts in the outer nuclear layer (white arrows). (Bottom Left) Optical coherence tomography scan of the same eye after 3 monthly intravitreal bevacizumab injections, microcystoid macular abnormalities resolved. (Top right) Inferior branch retinal vein occlusion with underlying severe primary open angle glaucoma. At baseline, optical coherence tomography scan shows microcystoid macular abnormalities nasally to the fovea (white arrows) and pseudocysts in the outer nuclear layer (white arrows). (Bottom right) Optical coherence tomography scan of the same eye after 3 monthly intravitreal bevacizumab injections, despite significant reduction of outer nuclear layer pseudocysts (white arrows), microcystoid macular abnormalities persisted (white arrowheads)

In our study, eyes with glaucoma required a greater number of anti-VEGF injections. As described previously, the clinical efficacy of anti-VEGF therapy for the treatment of RVO based CME is conclusive [3–6]. However, intravitreal anti-VEGF therapy may be less effective for the treatment of microcystoid abnormalities in eyes with underlying optic neuropathy, as highlighted in Fig. 5. Of note, the final BCVA (20/80) was worse in the group with GON compared to the non-glaucoma group (20/40) despite the fact that eyes in the glaucoma group received a greater number of intravitreal injections. The results presented in this paper highlight the importance of identifying microcystoid macular degeneration to prevent unnecessary therapy.

It is unclear if anti-VEGF therapy may have any visual benefit in eyes with predominantly microcystoid degeneration. In eyes with glaucoma, practitioners should assess for the presence of microcystoid macular abnormalities that are likely related to retrograde maculopathy and may represent an adverse clinical finding associated with poorer visual outcomes and which may be more resistant to anti-VEGF injection, requiring less aggressive therapy.

At present, there is no consensus term to refer to the presence of microcystoid degeneration in the INL. Previous papers have referred to these abnormalities them as ‘‘microcystic macular edema’’, [10, 16] ‘‘microcystic macular changes,”[14, 25] or “microcystoid macular changes” [15]. We adopted the the term ‘‘microcystoid macular abnormalities’’ as it better represents the abnormal anatomical findings identified with OCT scans. Nonetheless, a standardized nomenclature may be needed for use in future publications.

This study is the first, to our knowledge, to investigate the influence that glaucoma may have on the outcome of RVO. However, it was a retrospective analysis, and all patients were treated by the same retina specialist which may limit data heterogeneity and applicability to larger populations. Further, this study may overestimate the number non-exudative microcystoid macular abnormalities, since those microcysts located in the INL within areas of non-perfused retina could present a non-exudative appearance due to lack of flow. Identification of the slit like ellipsoid lesions can be challenging even with high-resolution cross-sectional SD-OCT but may be enhanced with en-face OCT in future studies. Since BRVO and CRVO have different clinical pattern, it would be more appropriate to analyze them separately; nevertheless, sample size was somewhat limited when analyzing BRVO and CRVO individually. Of note, the OCT volume scan was not dense, and therefore intervening areas of the overlay were not analyzed, which may have influenced the total number of microcystoid lesions counted. However, strengths of this study included the number of eyes with microcystoid degeneration with and without glaucoma, the extended follow-up period, and the comprehensive quantitative analysis of microcystoid lesions measured in 9 different quadrants of the macula.

In summary, the present study described a higher prevalence and number of microcystoid macular abnormalities in eyes with RVO and concomitant GON. Glaucomatous eyes required a greater number of intravitreal anti-VEGF injections and exhibited a worse baseline and final BCVA. Persistent microcystoid macular abnormalities despite treatment with anti-VEGF agents can be related to retrograde maculopathy and might not require additional treatment. Future prospective studies may address whether glaucoma would be associated with a higher prevalence of microcystoid degeneration in eyes with retinal vein occlusion.
